# The double stranded RNA analog poly-IC elicits both robust IFN-λ production and oncolytic activity in human gastrointestinal cancer cells

**DOI:** 10.18632/oncotarget.26121

**Published:** 2018-10-02

**Authors:** Chantal Bou-Hanna, Anne Jarry, Jean-François Mosnier, Céline Bossard, Christian L. Laboisse

**Affiliations:** ^1^ University of Nantes, EA4273 Biometadys, Nantes, France; ^2^ Current address: CRCINA, INSERM, Université d’Angers, Université de Nantes, Nantes, France; ^3^ Pathology Department, Nantes University Hospital, Nantes, France

**Keywords:** dsRNA/poly-IC, IFN-λ, immunoadjuvant, oncolysis, human gastrointestinal cancer

## Abstract

**Purpose:**

Type III IFN (IFN-λ) is the dominant frontline response over type I IFN in human normal intestinal epithelial cells upon viral infection, this response being mimicked by the dsRNA analog poly-IC. Poly-IC also induces cell death in murine intestinal crypts *ex vivo*. Here we examined whether these innate defense functions of normal intestinal epithelial cells are recapitulated in gastrointestinal carcinoma cells so that they could be harnessed to exert both immunoadjuvant and oncolytic functions, an unknown issue yet.

**Experimental design:**

Four human gastrointestinal carcinoma cell lines versus the Jurkat lymphoma cell line were used to assess the effects of intracellular poly-IC on i) IFN-λ secretion and cell proliferation and ii) role of NFκB signaling using the NFκB inhibitory peptide SN50 as a screening probe and a siRNA approach.

**Results:**

Poly-IC induced in all cell lines except Jurkat both a robust IFN-λ secretion and a cytoreductive effect on adherent cells, restricted to proliferating cells and associated with cellular shedding and reduced clonogenicity of the shed cells. Collectively these findings demonstrate the oncolytic activity of poly-IC. Inhibiting NFκB in T84 cells using a siRNA approach decreased IFN-λ production without protecting the cells from the poly-IC oncolytic effects. In line with these findings IFN-λ, that upregulated the anti-viral protein MxA, was unable per se to alter T84 cell proliferation.

**Conclusion:**

Our demonstration that poly-IC-induced concomitant recapitulation of two innate functions of normal intestine, i.e. IFN-λ production and cell death, by human gastrointestinal cancer cells opens new perspectives in gastrointestinal cancer treatment.

## INTRODUCTION

The gastrointestinal epithelial lining is the first line of defense against potentially noxious agents including bacteria, bacterial products and viruses. Based on both primary cultures of normal human intestinal epithelial cells [1] and on *ex vivo* explant cultures of human normal colonic mucosa we have contributed to the emergence of the concept of innate immune functions as well as immunomodulatory functions of the human intestinal mucosa epithelium [2–5].

Interestingly, the innate antiviral response of epithelial cells involves interferon (IFN) production, namely type I (α/β), and type III (λ) IFN. While a huge literature covers type 1 IFN production and effects, there is still much to understand about the more recently identified type 3 IFN [6] that consist in 3 members IFN- λ-1 to 3. Both types l and lll interferon genes possess NF-κB binding sites, essential for gene activation by viruses [7, 8]. IFN-λ interacts and signals through a unique heterodimeric receptor complex consisting of IFN-λ receptor 1 and the IL-10 receptor subunit 2 [9]. However, unlike the receptors for type I IFNs, which are broadly expressed on virtually all cell types, IFN-III receptors exhibit a more restricted tissue distribution [6, 10]. Because of the use of distinct receptors, types I and III IFNs likely do not signal identical biological outcomes in anti-viral and anti-cancer activities [6]. The activity of IFN-λ is highly prominent in barrier epithelia compared with other cell types [11]. In addition IFN-λ has lower toxicity than IFN-α [12]. Interestingly IFN-λ has been recently shown to exert antitumor effects in both murine and human models. This has been shown to occur through direct effects on target tumor cells as well as through indirect-immune-mediated responses [13, 14].

Recently human intestinal enteroids (HIEs) that exhibit a similar cellular composition to the intestinal epithelium have been established, and used to study viral epithelial interactions [15]. Using this model system Saxena *et al.* have shown that rotavirus infection of human intestinal epithelial cells induces type III IFN as the dominant transcriptional response over type 1 IFN [16]. Such a conclusion was also reached by Pervolaraki *et al.* [17] who state that type III IFN is the frontline of antiviral response in the human gut. Interestingly viroplasm-free dsRNA is present in the cytoplasm of rotavirus-infected cells and is a key intermediate in the replication cycle of many viruses, including other major human enteric viral pathogens [16, 18]. In this context, it is worth noting that the type III IFN response to rotavirus was also obtained using the dsRNA analog poly-IC [16]. Finally, it can be speculated that human intestinal epithelial cells are programmed to respond to viral dsRNA with type III IFN [16].

Other experiments have shown that the dsRNA analog poly-IC induces crypt cell death in murine enteroids [19]. In the same way poly-IC administered to mice induced intestinal epithelial cell death within a few hours (3 to 6 h) [20]. Apoptotic deletion of infected epithelial cells translates into pathological cell shedding [21].

Taken together, these findings strongly suggest that the dsRNA analog poly-IC is able to trigger a dual effect in normal intestinal cells, i.e. an immunoadjuvant effect represented by IFN-λ production and epithelial cell shedding.

In this context, we hypothesized that human gastrointestinal carcinoma cells could maintain these dual functions upon intracellular treatment by the dsRNA analog poly-IC. Our aim was twofold: i) determine concomitantly both IFN-λ secretion and cell proliferation/shedding upon poly-IC treatment in several human gastrointestinal carcinoma cell lines; and ii) evaluate whether these two parameters are connected via a common pathway using NFκB signaling as a probe.

## RESULTS

### Intracellular poly-IC induces IFN-λ production in human gastrointestinal cancer cell lines

**Figure 1:**
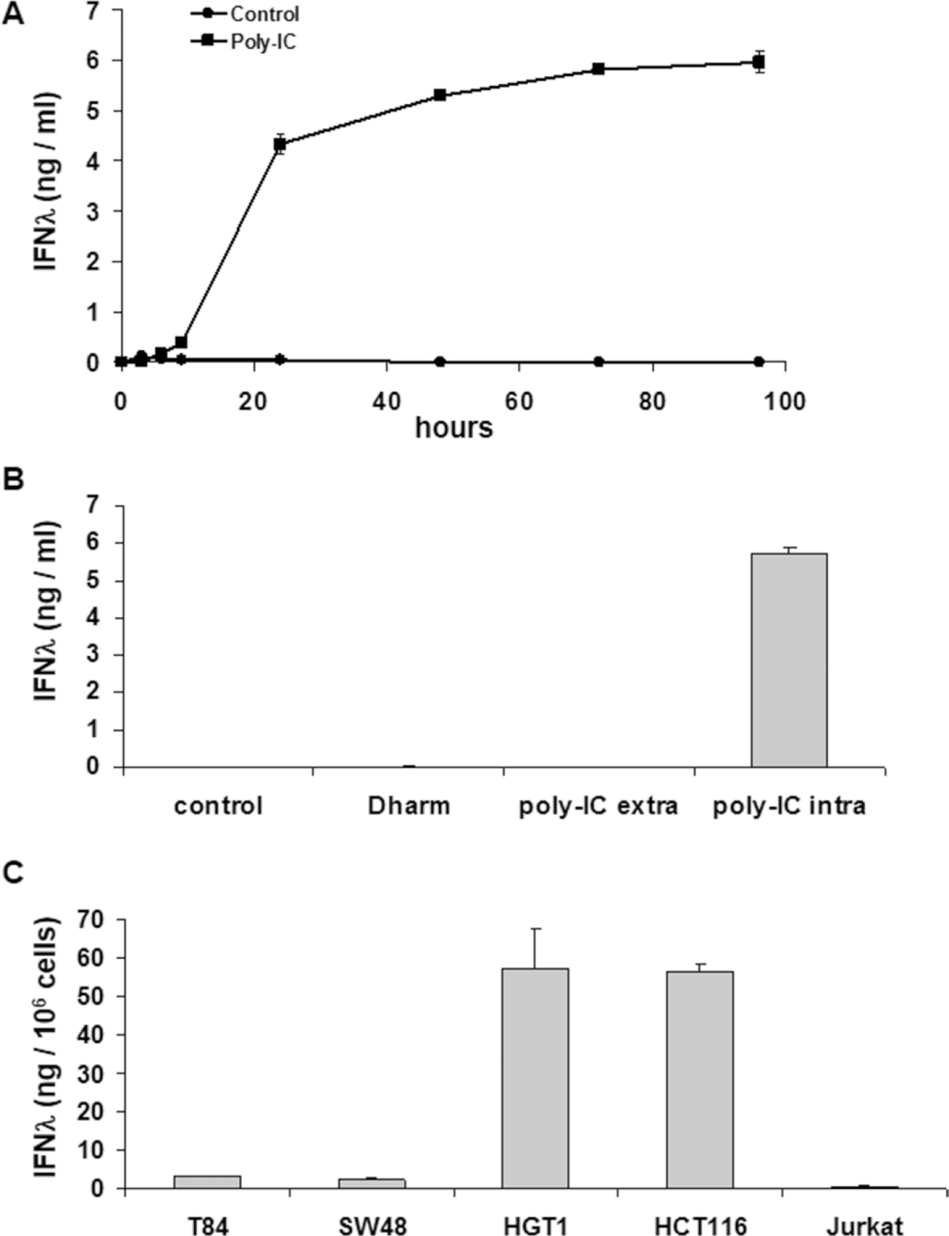
Intracellular Poly-IC elicits IFN-λ production in gastrointestinal cancer cell lines as measured by ELISA in culture supernatants (**A**) Time-dependent effect of intracellular Poly-IC on T84 cells. Proliferating T84 cells, maintained in 6-well plates, were treated for the indicated time points with 0.64 μg/ml poly-IC in presence of Dharmafect (intracellular Poly-IC). Each symbol represents the mean ± sem of 3 experiments performed in triplicate. (**B**) T84 cells were incubated with extracellular (extra) or intracellular (intra) poly-IC (0.64 μg/ml) for 72 h, or with medium (control) or vehicle alone (Dharm). Mean ± sem of 3 experiments performed in triplicate. (poly-IC intra vs Dharm: *p* < 0.0001; poly-IC extra vs control: NS). (**C**) Gastrointestinal cell lines or Jurkat cells were treated with intracellular Poly-IC for 72 h. Mean ± sem of 3 experiments performed in triplicate.

As shown in Figure 1A, T84 cancer cells exposed intracellularly to Poly-IC produced huge amounts of IFN-λ in a time-dependent manner. The kinetics of IFN- λ production shows two phases: a steep rise in IFN-λ accumulation in the medium, significant at time point 6 h, peaking at 72 h and followed by a plateau up to 96 h. In addition, IFN-λ production was almost undetectable when T84 cells were treated with extracellular poly-IC for 72 h (Figure 1B).

We then determined the kinetics of committment to IFN-λ production. To this end, a variable exposure time to poly-IC (3 h, 6 h, 9 h) was followed by replacement of the poly-IC-containing medium by fresh medium. The read-out of results was the determination of IFN-λ concentration at time point 72 h. These experiments showed that an exposure time of 3 h to poly-IC was sufficient to commit the cells to a significant IFN-λ production.

We then extended the evaluation of the effects of intracellular poly-IC to several human cancer cell lines of gastrointestinal origin or not (Jurkat). As shown in Figure 1C, all human gastrointestinal cancer cell lines produced huge amounts of IFN-λ upon a 72 h exposure to intracellular poly-IC, with large variation across cell lines. Interestingly the Jurkat cells were insensitive to poly-IC.

### Differential subordination of poly-IC-induced IFN-λ production to SN50

**Figure 2:**
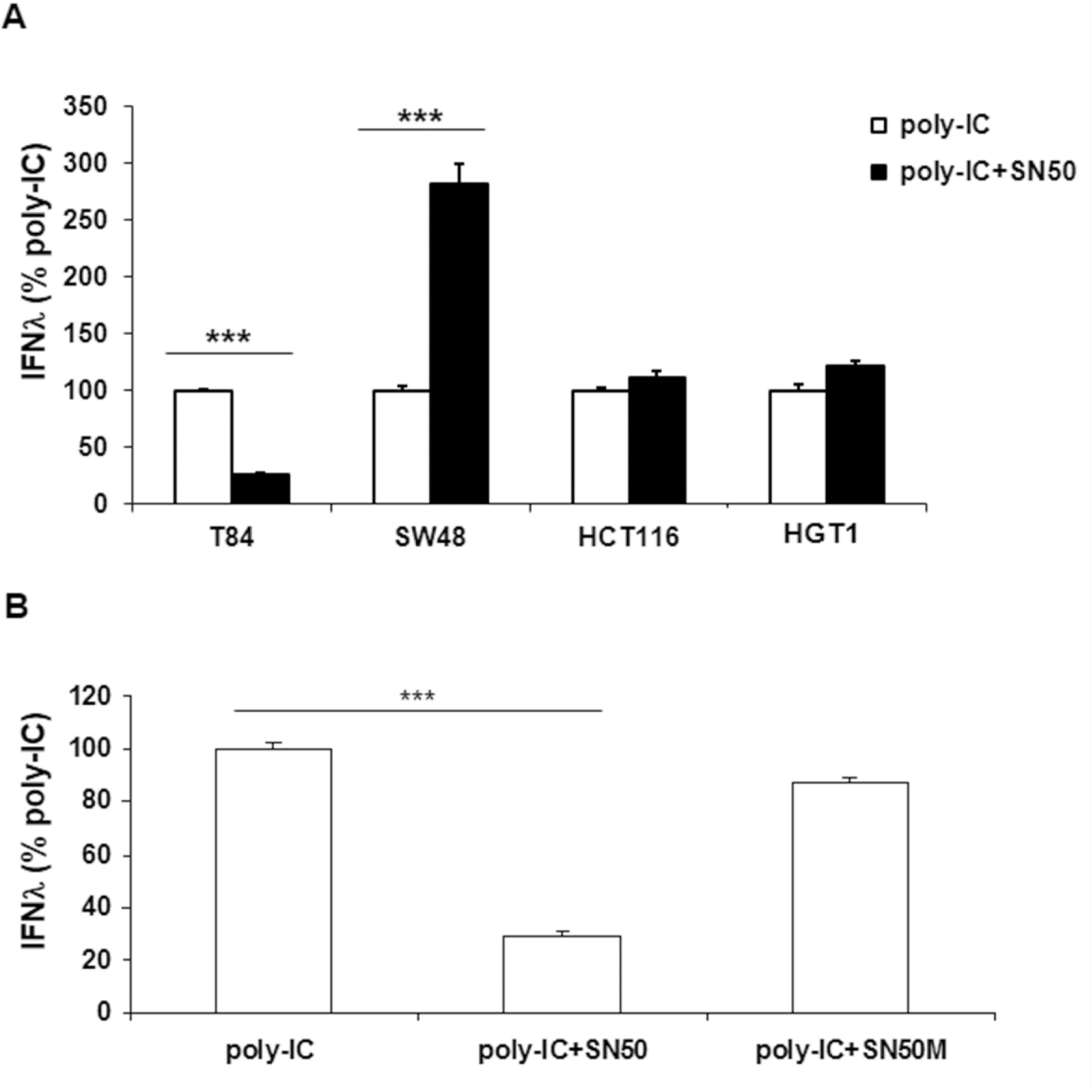
Differential effects of SN50 on Poly-IC-induced IFN-λ production in gastrointestinal cancer cell lines (**A**) Various gastrointestinal cell lines were treated for 18 h with intracellular Poly-IC (0.64 μg/ml) in presence or absence of the NFκB nuclear translocation inhibitor SN50 (80 μg/ml). (**B**) T84 cells were treated for 18 h with intracellular Poly-IC in presence or absence of SN50 or the control peptide SN50M (80 μg/ml). IFN-λ was measured by ELISA in culture supernatants. Results are expressed as the percentage of IFN-λ production upon Poly-IC treatment. Mean ± sem of 2 experiments performed in triplicate. ^***^p < 0.0001.

NFκB signaling is known to mediate IFN type 1 production upon cell exposure to poly-IC. It is the reason why we examined the effect of SN50, a cell permeable peptide that inhibits the translocation of p50/RelA (p65) into the nucleus. As expected from a NFκB-dependent mechanism, the poly-IC-induced IFN-λ production was significantly inhibited by SN50 in T84 cells (Figure 2A). By contrast SN50 synergized the effect of poly-IC on SW48 cells. SN50 had no effect on poly-IC-induced IFN-λ production in HGT-1 and HCT116 cells (Figure 2A). We verified that SN50 alone had no effect on the cultured cells. In addition, the control peptide SN50M did not modify significantly the secretory response of T84 to poly-IC (Figure 2B). Altogether, these results led to two conclusions: i) there is a differential sensitivity of the various gastrointestinal cell lines to SN50; ii) this pharmacological approach strongly suggests that the IFN-λ secretory response to poly-IC is mediated by NFκB signaling in T84 cells.

### Evidence for NFκBp65 signaling in poly-IC-induced IFN-λ production in T84 cells

**Figure 3:**
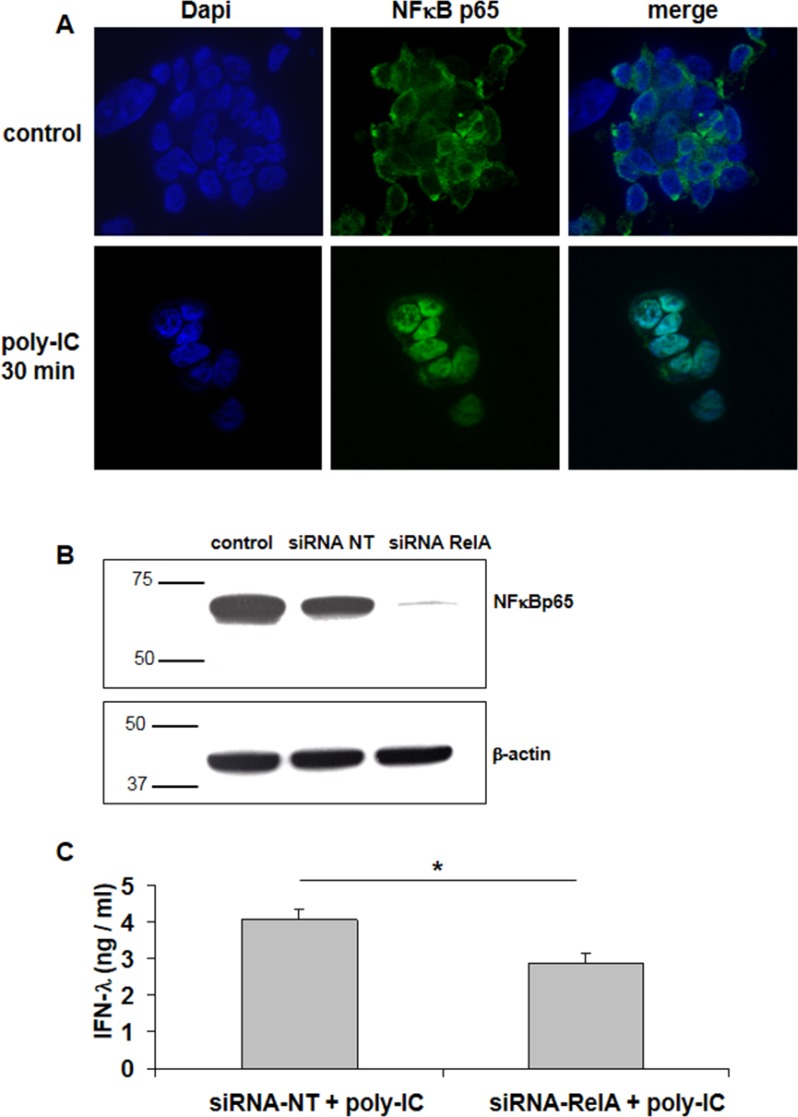
Involvement of NFκBp65 (RelA) signaling in Poly-IC-induced IFN-λ production in T84 cells (**A**) Immunofluorescence staining with an antibody directed to NFκBp65 subunit. T84 cells, cultured on Labtek chambers, were treated or not with intracellular Poly-IC for 30 min. Poly-IC induced an early cytoplasmic to nuclear translocation of NFκBp65 (green). Nuclei were stained with Dapi (blue). Original magnification × 630. (**B**) Immunoblot analysis of NFκBp65 and of the loading control β-actin, of T84 cells treated with siRNA non target (NT) or siRNA RelA for 48 h. Numbers on the left indicate molecular weight markers. Immunoblot representative of 4 different experiments. (**C**) Effect of siRNA RelA on poly-IC-induced IFN-λ production. T84 cells were treated for 48 h with siRNA NT or siRNA RelA and then with poly-IC + Dharmafect for 18 h. IFN-λ was measured by ELISA in culture supernatants. Mean ± sem of 2 experiments performed in triplicate. ^*^p = 0.03.

Consistent with the concept that NFκB signaling involves the activation of a preexisting complex sequestered in the cytoplasm, we found that a 30 min incubation with poly-IC was sufficient to induce the nuclear translocation of NFκBp65 (Figure 3A).

We then used a siRNA approach to confirm the involvement of NFκB in poly-IC-induced IFN-λ production in T84 cells. As shown in Figure 3B, a siRNA directed to p65 (siRNA RelA) abolished the expression of NFκBp65. In addition Poly-IC-induced IFN-λ production was significantly decreased in siRNA RelA-treated cells (Figure 3C).

### Oncolytic activity of intracellular poly-IC

**Figure 4:**
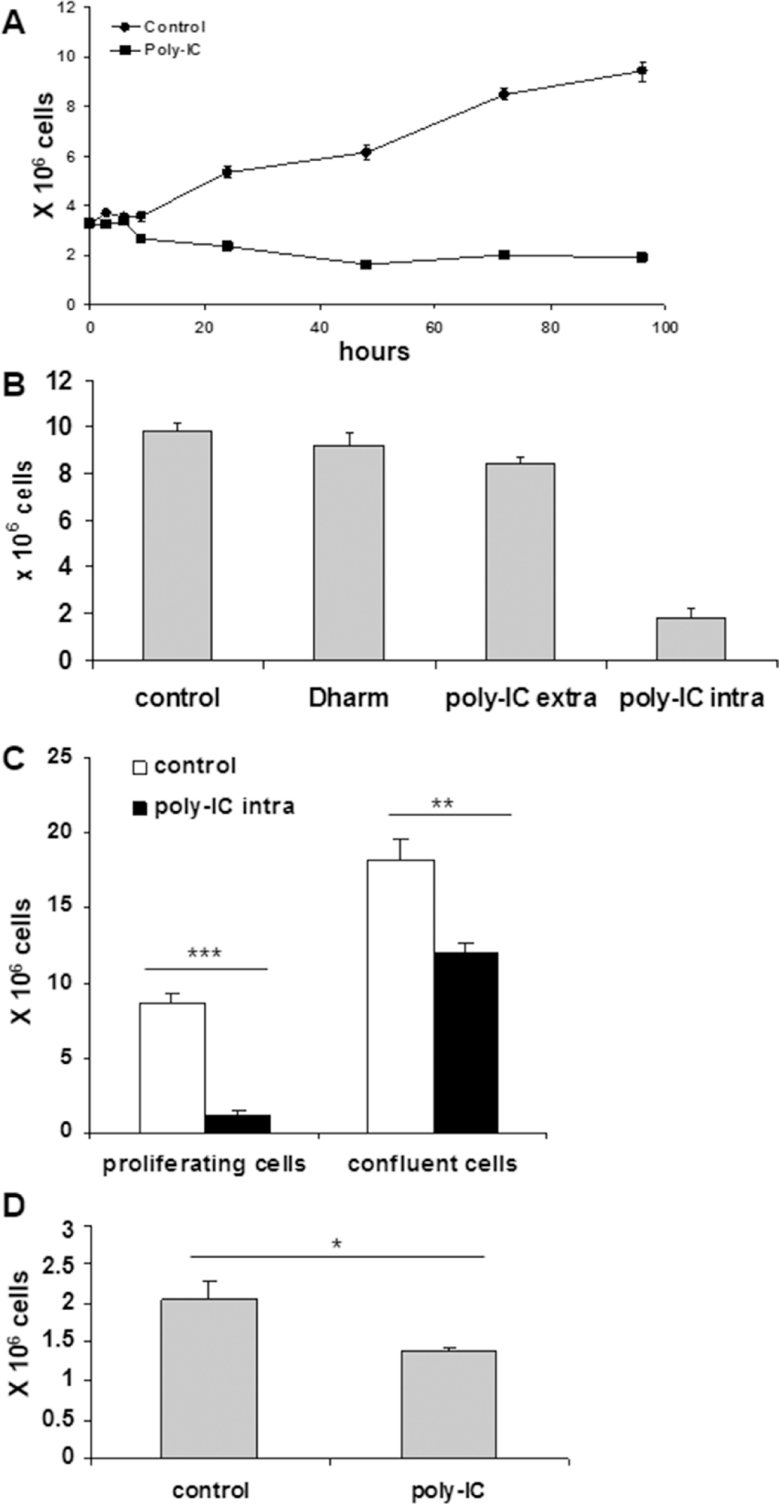
Intracellular Poly-IC induces an antiproliferative/cytoreductive effect on T84 cells (**A**) Time-dependent effect of intracellular Poly-IC on cell growth. Proliferating T84 cells, maintained in 6-well plates, were submitted or not to poly-IC 0.64 μg/ml) for indicated time points and then detached with trypsin and counted. Each symbol represents the mean ± sem of 3 experiments performed in triplicate. (Poly-IC vs control: *p =* 0.003 at 9 h; *p* < 0.0001 from 12 h to 96 h). (**B**) T84 cells were incubated with extracellular (extra) or intracellular (intra) poly-IC or with medium (control) or vehicle alone (Dharm) for 72 h, then detached with trypsin and counted. (poly-IC intra vs Dharm: *p* < 0.0001; poly-IC extra vs control: NS). (**C**) Proliferating or postconfluent T84 cells were treated with intracellular Poly-IC for 72 h, then detached with trypsin and counted. (**D**) Remaining adherent cells of the Poly-IC-treated postconfluent T84 cells were detached and seeded in fresh medium for 4 days. At end of incubation, cells were detached and counted. B to D: mean ± sem of 3 experiments performed in triplicate. ^*^p = 0.01; ^**^p = 0.003; ^***^p < 0.0001.

As shown in Figure 4A, intracellular poly-IC induced a cytoreductive/antiproliferative effect over time associated with the shedding of numerous cells into the medium. Interestingly, a significant cytoreduction was observed as early as 9 h incubation with poly-IC. This effect was not observed when T84 cells were exposed to extracellular poly-IC (Figure 4B).

We then determined the kinetics of commitment to cytoreduction. To this end a variable exposure time of T84 cells to poly-IC (3 h, 6 h, 9 h) was followed by replacement of the poly-IC-containing medium by fresh medium. The evaluation of the adherent cell number was the read-out at time point 72 h. Interestingly the commitment time for the cytoreduction was 6 h, i.e. a longer duration than that necessary for IFN-λ production.

In a tumor, a large fraction of cells are in a quiescent state. It was therefore important to examine the action of poly-IC in postconfluent cells versus actively proliferative cells. As shown in Figure 4C, post-confluency afforded a significant protection to a 72 h exposure to poly-IC, as the percentage of adherent viable cells dropped from 67% in confluent T84 cultures to 13% in exponentially growing cultures. Interestingly, when the remaining adherent cells of the poly-IC-treated postconfluent cultures were dissociated and seeded in fresh medium, a delayed cytoreductive effect was noted (Figure 4D).

**Figure 5:**
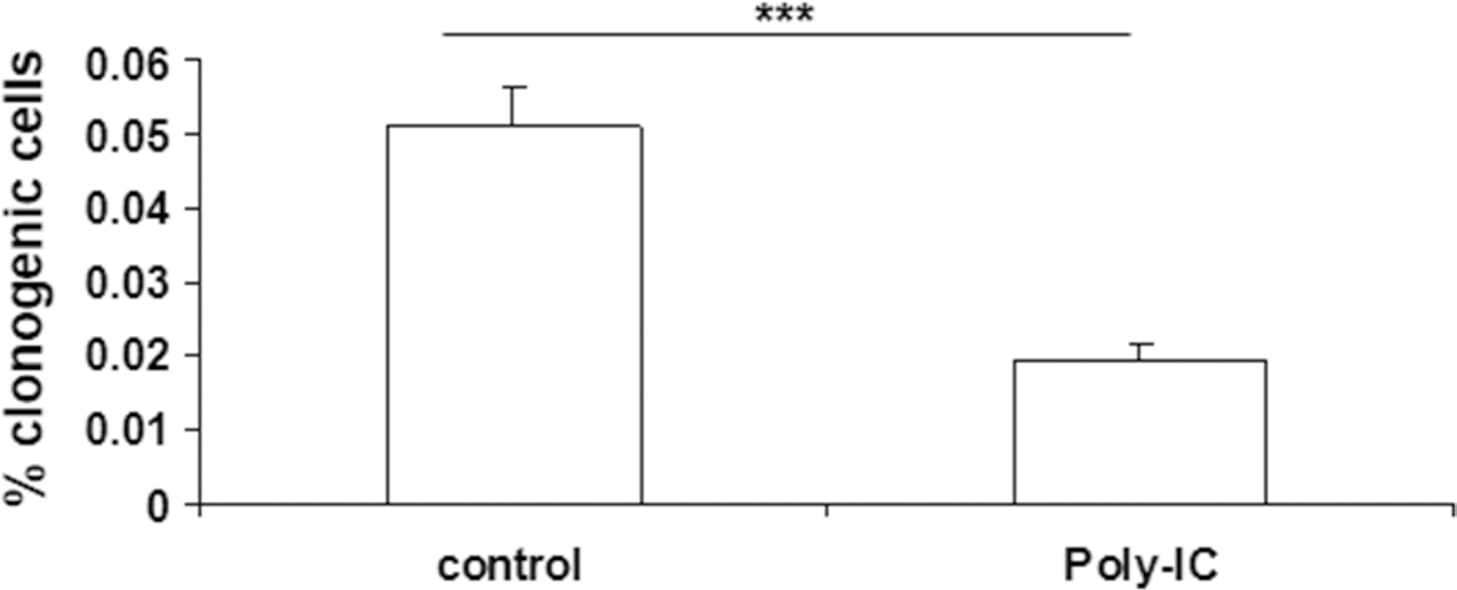
Poly-IC decreased the number of clonogenic T84 cells After a 72 h treatment of proliferating T84 cells with Poly-IC, the floating cells were seeded in fresh medium at a clonogenic density (see Materials and Methods). The colonies were counted after a 10-day culture. Mean ± sem of 2 separate experiments with 15 control flasks and 15 Poly-IC-treated flasks per experiment. ^***^p = 0.0001.

As exposure to poly-IC led to a huge cellular shedding it was important to determine the clonogenic potential of the shed cells. To this end, the cells floating in the medium were collected after a 72 h treatment with poly-IC and seeded in fresh medium in 75 cm^**2**^ flasks. As shown in Figure 5, the clonogenicity of the poly-IC-treated T84 cells was significantly lower than that of control cells. Together, the cytoreductive activity and the decreased clonogenicity of the shed cells support the concept of an oncolytic effect of intracellular poly-IC.

**Figure 6:**
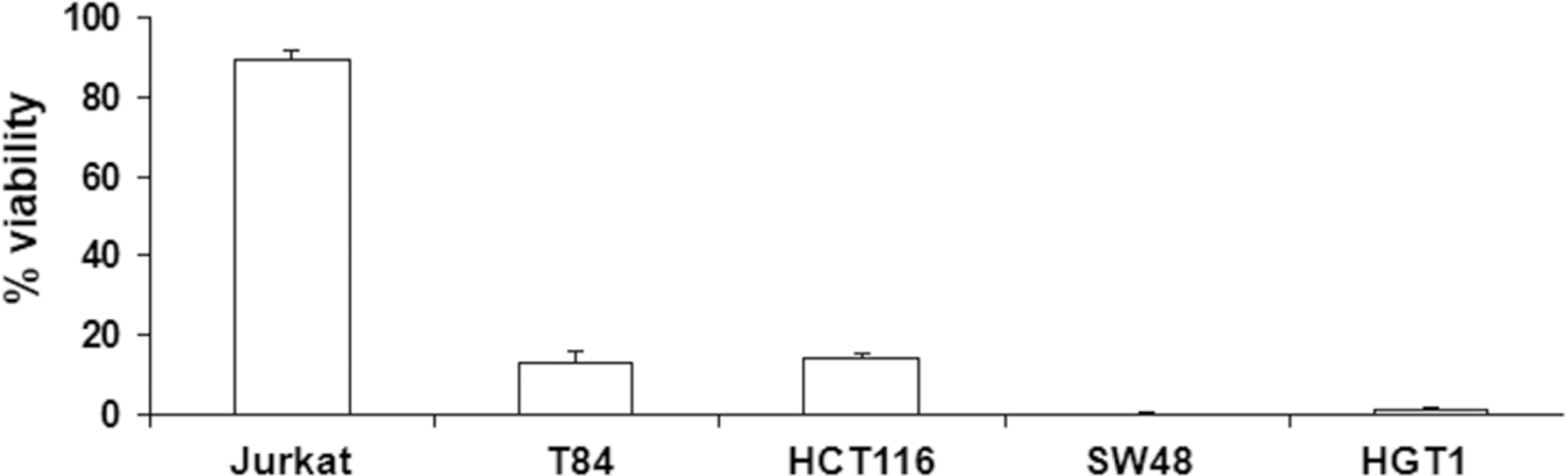
Antiproliferative/cytoreductive effect of intracellular Poly-IC on various cancer cell lines Proliferating gastrointestinal cell lines or Jurkat cells were treated or not with intracellular Poly-IC for 72 h. Gastrointestinal cells were then detached with trypsin and counted; Jurkat cells, growing in suspension, were directly counted. Results are expressed as percentage of viability versus control, untreated cultures. Mean ± sem of 3 experiments performed in triplicate.

As shown in Figure 6, poly-IC exerted a cytoreductive effect in all gastrointestinal cancer cell lines and not in Jurkat cells.

### Effects of SN50 and siRNAp65 on the cytoreductive effect of poly-IC

**Figure 7:**
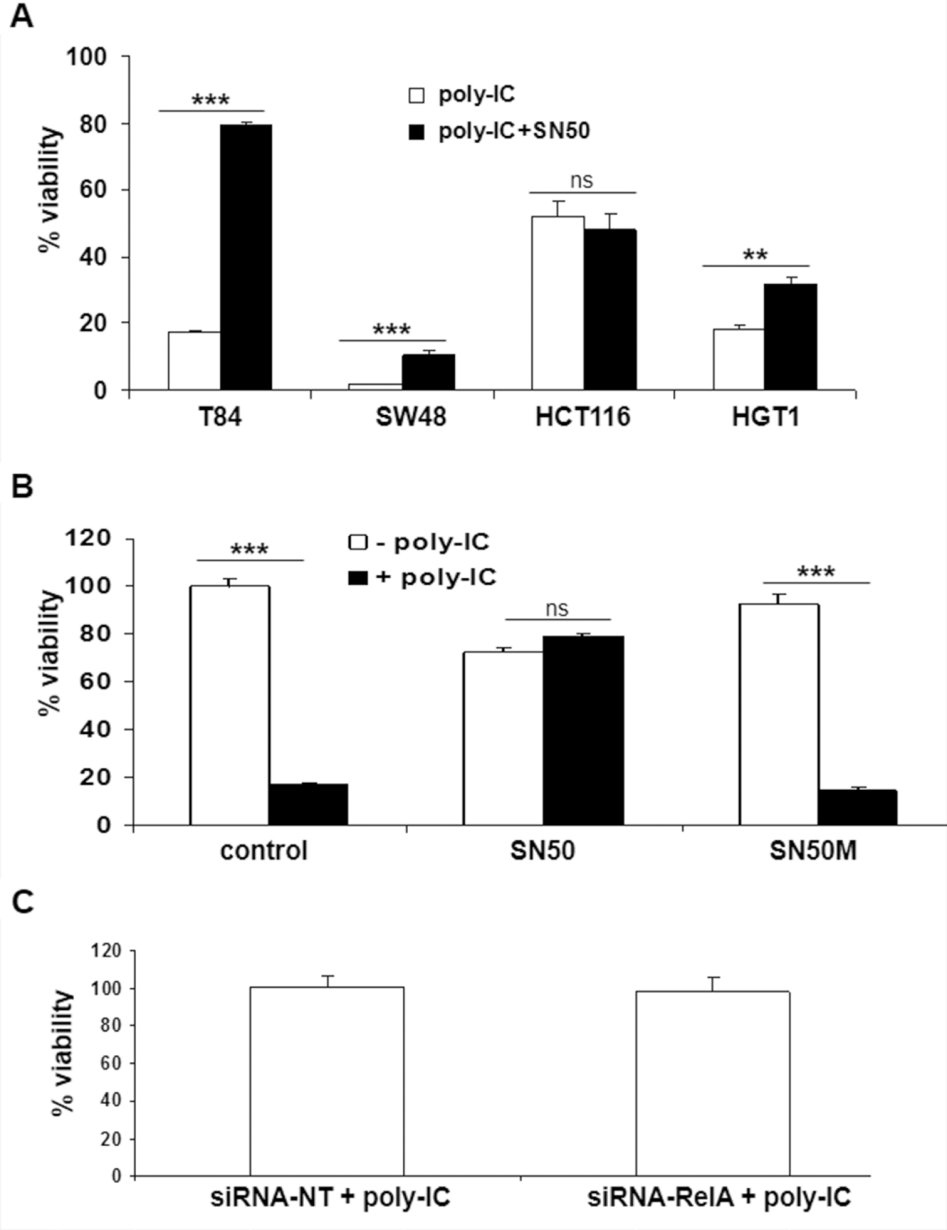
Effect of SN50 and siRNA RelA on the antiproliferative/cytoreductive effect of Poly-IC (**A**) The various gastrointestinal cancer cell lines were treated with intracellular Poly-IC in the presence or absence of SN50 for 18 h. (^**^p = 0.003; ^***^p < 0.0001). (**B**) T84 cells were treated or not with intracellular Poly-IC in the presence or absence of SN50 or of the control inhibitor SN50M (both 80 μg/ml) for 18 h (poly-IC + SN50M vs poly-IC: NS whereas poly-IC + SN50 vs poly-IC: *p* < 0.0001). In A and B, Results are expressed as percentage of viability versus control cultures. Mean ± sem of 2 experiments performed in triplicate. (**C**) T84 cells were treated with siRNA NT or siRNA RelA for 48 h, then allowed to recover in fresh medium for 24 h, and then treated with intracellular poly-IC for 18 h. Results are expressed as percentage of poly-IC + siRNA NT, considered as 100%. Mean ± sem of 2 experiments performed in triplicate (siRNA-RelA vs siRNA-NT: NS).

SN50 blocked the cytoreductive effect of poly-IC in T84 cells (Figure 7A). In addition, a weak but significant protective effect of SN50 was observed in SW48 and HGT-1 cells. HCT116 cells were insensitive to SN50. In T84 cells, the control peptide SN50M did not afford any protection against poly-IC cytoreductive effect (Figure 7B). Interestingly, the siRNA RelA did not modify the cytoreductive effect of poly-IC on T84 cells (Figure 7C), while in these experimental conditions it led to a significant decrease of IFN-λ production (Figure 2A).

Together these results show i) the heterogeneity of the cellular response to SN50 across the gastrointestinal cancer cell lines upon poly-IC treatment; and ii) the absence of subordination of the cytoreductive effect of poly-IC to NF-κBp65 in T84 cells.

### Effects of IFN-λ on T84 cell proliferation

**Figure 8:**
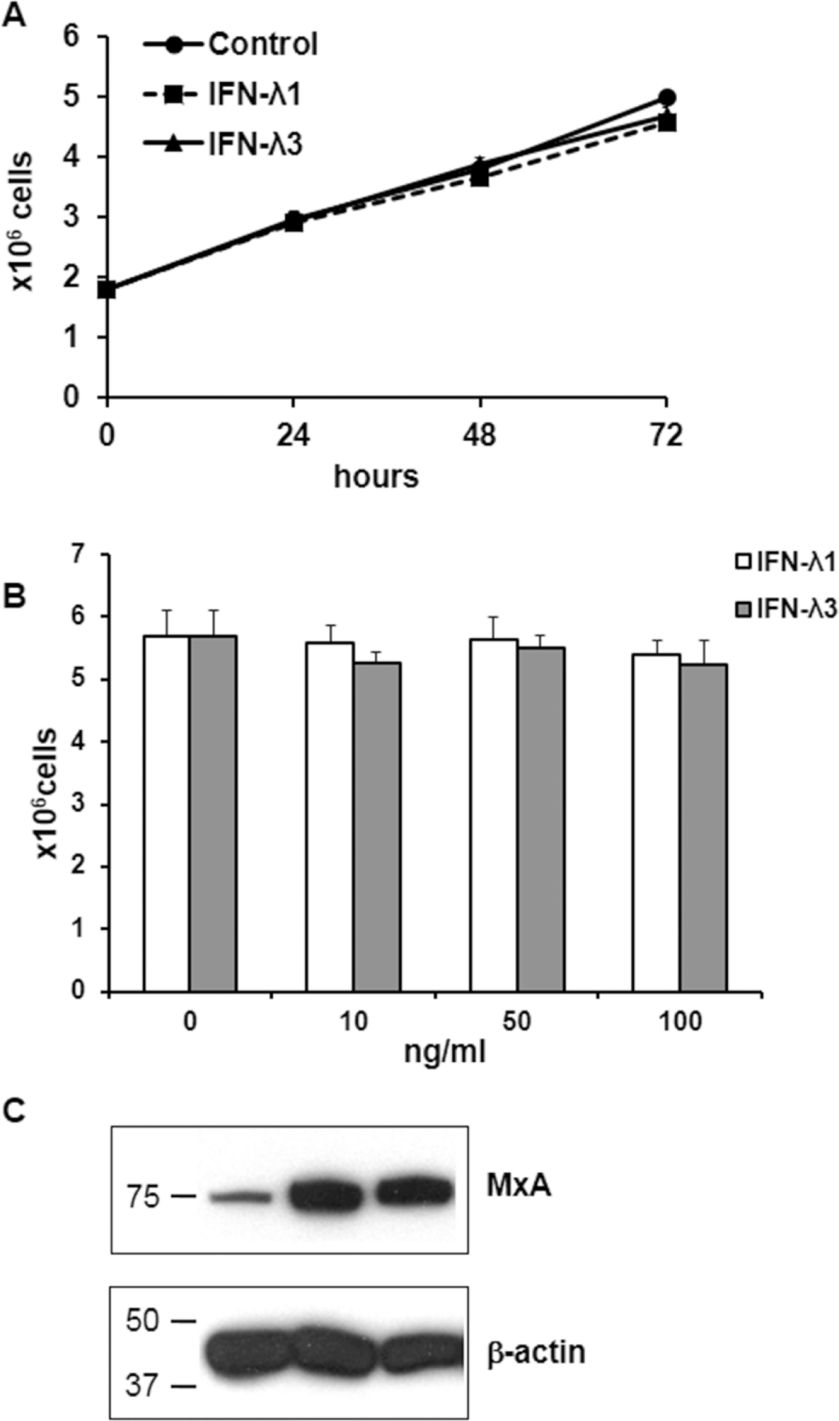
Effects of IFN-λ on T84 cell proliferation and MxA expression (**A**) Time-dependent effect of IFN-λ on T84 cells. Proliferating T84 cells were incubated or not in the presence of IFN-l1 or IFN-l3 (10 ng/ml) for the indicated time points. They were then detached with trypsin and counted. Each symbol represents the mean ± sem of 3 experiments performed in triplicate. Statistical analyses were performed using a student *t*-test (no statistical difference). (**B**) dose-response effect of IFN-λ on T84 cells. Proliferating T84 cells were incubated for 72 h in the presence of increasing doses of IFN-λ1 or IFN-λ3. At the end of incubation they were detached with trypsin and counted. Bar graph representation of the mean ± sem of 3 experiments performed in triplicate. Statistical analyses were performed using a student *t*-test (no statistical difference). (**C**) Immunoblot analysis of MxA and of the loading control β-actin of T84 cells incubated for 72 h with or without IFN-λ1 or IFN-λ3. Numbers on the left indicate molecular weight markers.

We next examined whether IFN-λ was able *per se* to decrease T84 cell proliferation. Treatment of T84 cells at various time points (24 h, 48 h, 72 h) with IFN-λ1 (IL-29) or IFN-λ3 (IL-28) used at a concentration of 10 ng/ml, the maximal concentration released upon poly-IC (see Figure 1), did not alter cell proliferation compared with controls (Figure 8A). There was also no change in the number of shed cells. In addition, exposure of T84 cells to increasing doses of IFN-λ1 or IFN-λ3 (10, 50, 100 ng/ml) for 72 h did not modify cell proliferation (Figure 8B). To verify the existence of functional IFN-λ receptors in T84 cells, we examined the expression of the anti-viral protein MxA, known to be induced by IFN-λ in normal epithelial cells. As shown in Figure 8C, IFN-λ1 and IFN-λ3 upregulated MxA expression level in T84 cells.

## DISCUSSION

Based on histopathological analysis, gastrointestinal carcinomas can display histological features of their tissue of origin, e.g. gland formation, or mucus production. Moreover, using gastrointestinal carcinoma cell lines, it is clear that the differentiated phenotype includes not only morphological, but also functional characteristics subordinated to the same control mechanisms as normal cells [22–26]. However, as to whether the innate defense functions of human normal gastrointestinal epithelial cells are recapitulated in carcinoma cells has remained poorly explored. This study brings out the first demonstration of a dual effect of the dsRNA analog poly-IC on human gastrointestinal carcinoma cells, through the induction of IFN-λ secretion and oncolytic effects. Our finding of the subordination of this dual effect to the intracellular presence of poly-IC is in line with the concept of recapitulation by carcinoma cells of an innate program of defense against virally-infected epithelial barrier cells.

In fact, type III IFN/IFN-λ is now known as the dominant immunoadjuvant response over type I IFN upon viral infection or poly-IC treatment in human normal intestinal epithelial cells [15, 16]. However, the biological significance of the transcriptional activation of IFN-λ by intracellular poly-IC has remained unsettled. Indeed, Saxena *et al.* [15] showed a major transcriptional IFN-λ response to dsRNA with low protein secretion, while Pervolaraki *et al.* [17] showed a mRNA response and subsequent huge protein secretion. Thus we decided to focus on IFN-λ production at the protein level which is the biologically important endpoint. Interestingly, a huge secretion of IFN-λ was found in all the gastrointestinal carcinoma cell lines tested with a relative heterogeneity across them. At difference with our experimental protocol, Swider *et al.* [27] exposed colon cancer cells to a very high (50 μg/ml) concentration of extracellular poly-IC that resulted paradoxically in a several orders of magnitude lower production of IFN-λ than that we obtained with intracellular poly-IC delivery. Together these findings are in line with the concept that only the transfected poly-IC is able to trigger an IFN-λ response. Furthermore, the IFN-λ concentrations we observed (1–8 ng/ml) were compatible with a biological effect of IFN-λ. In fact, such concentrations were highly efficient in stimulating the anti-viral genes MxA and OAS in a biological assay [28]. In addition, upregulation of IFN-λ-stimulated genes was observed in primary human enteroids in a dose-dependent manner, starting at around 10 ng/ml IFN-λ [17]. Finally, in line with the concept that IFN-λ production is a feature of barrier epithelia, either normal or tumorous, the lymphoid Jurkat cell line, known to develop a Th2 response upon dsRNA treatment [29], was unable to produce IFN-λ upon intracellular poly-IC.

The assessment of cell proliferation as determined by counting viable adherent cells and examination of culture dishes with the inverted microscope showed that poly-IC triggered cell shedding associated with a cytoreductive effect. This pattern is reminiscent of epithelial cell shedding of infected cells [20, 21]. At that point, it was necessary to determine the biological status of the shed cells. Our demonstration of both a cytoreductive effect of poly-IC on adherent cells and a decreased clonogenicity of shed cells argue for an oncolytic activity of intracellular poly-IC. Noticeably, we showed that poly-IC oncolysis occurs mainly on proliferative cells. Quiescent post-confluent cells were relatively tolerant to intracellular poly-IC and regained vulnerability upon regrowth even in the absence of the oncolytic agent. The effect of tumor cell quiescence, an escape mechanism towards chemotherapeutic agents, would be limited in case of cancer treatment with poly-IC.

We next showed that the oncolytic effect of poly-IC is not subordinated to IFN-λ production since: i) a commitment period as short as 3 h was enough to induce IFN-λ secretion without triggering any significant oncolysis; and ii) IFN-λ1 or IFN-λ3 had *per se* no oncolytic effect on T84 cells. Our findings are in line with those of Qu *et al.* [30] who showed that the apoptotic response to poly-IC of human gastric cancer cells is independent from type I IFN production. In addition, our demonstration of functional IFN-λ receptors on T84 cells is consistent with an autocrine effect of IFN-λ released upon poly-IC treatment, restricted to an innate immune defense mechanism without altering cell proliferation. As to whether this conclusion can be extrapolated to all gastrointestinal cancer cell lines remain to be determined.

Finally, we examined the existence of a hypothetic common intracellular signaling pathway for both IFN-λ production and oncolysis. NFκB activation was a likely candidate on two grounds: i) a rapid NFκB activation was shown upon exposure of human gastric adenocarcinoma cells to poly-IC [30]; and ii) NFκB signaling was involved in IFN-λ production upon viral infection [7, 8]. In our work, we observed a rapid (within 30 minutes) NFκB p65 nuclear translocation and the functional effects of NFκB inactivation were examined based on a pharmacological screening using SN50, known to inhibit NFκB nuclear translocation. Surprisingly, SN50 inhibited IFN-λ production only in T84 cells, a finding being confirmed by a siRNA-RelA approach. As NFκB activation has been shown to vary across human colonic cancer cell lines submitted to a given stress [31], it is likely that the poly-IC-induced IFN-λ response results from the activation of alternative pathways in various cell lines. As for the hypothetical involvement of NFκB activation in poly-IC-induced oncolysis, it is worth noting that several publications have shown a protective effect of SN50 upon stress-induced cell death [32–34] including poly-IC-induced cell apoptosis [35]. These findings based on the parallel observation of NFκB activation and protective activity of SN50 have led to the conclusion of deleterious effects of NF-kB activation. However, the absence of a genetic approach aimed at blocking NFκB activation should caution against such a conclusion. In our study, SN50 exerted a protective effect on poly-IC-induced cytotoxicity, not replicated by the siRNA-RelA approach. Altogether, our findings suggest that the protective effects of SN50 are off-target effects. In fact, SN50 peptide is now known to compete for several proteins involved in nuclear import [36]. Altogether, our findings suggest that in the T84 cell line, NFκB activation is involved only in poly-IC immunoadjuvant effect and not in its oncolytic effect. Finally, the fact that siRNA RelA decreases IFN-λ secretion without altering cell proliferation reinforces the concept that IFN-λ is not causally related to poly-IC-induced oncolysis. In addition, the homogeneity of the dual response of the four gastrointestinal carcinoma cell lines to poly-IC is underlied by a heterogeneity of intracellular signaling mechanisms. Finally, the apparent paradox of such a robust dual activity in the face of a heterogeneity of signaling pathways argues for a redundancy of these pathways.

In conclusion, it is well known that chemotherapies have only a suboptimal therapeutic effect in gastrointestinal advanced cancers. Therefore, an immunotherapy approach has been proposed in colorectal cancers, aimed at reactivating the anti-tumor immune response based on the blockade of checkpoints [37]. In addition, recent investigations show that the combination of immune checkpoint blockade with oncolytic vaccinia virus can work synergistically [38]. As oncolysis is subordinated to intracellular dsRNA generated in vaccinia virus infected cells [39], it can be speculated that direct dsRNA transfection to cancer cells could be a substitute to virotherapy in a combination strategy with an immune checkpoint inhibitor. In addition using poly-IC as an oncolytic agent could overcome the poor infection potential of oncolytic reovirus [40]. Here we bring out the proof of concept that poly-IC, via its oncolytic and immunoadjuvant effects, could be an innovative therapeutic strategy, as IFN-λ is known to reinforce the anti-tumor Th1 response [14], dampen the Th2 response [41] and activate NK/NKT cells tumor-killing activity in preclinical cancer models [42, 43]. Interestingly, the efficacy and safety of intratumoral poly-ICLC (Hiltonol) was shown first in a compassionate study in one patient [44] and very recently in a phase II clinical trial enrolling 8 patients [45].

## MATERIALS AND METHODS

### Cancer cell lines

The following human cell lines were used in this study: colonic cancer cell lines (T84, SW48, HCT116, purchased from ATCC), a gastric cancer cell line (HGT-1) [22] and a T-cell leukemia cell line (Jurkat, ATCC). Cell lines were maintained in DMEM/10% heat-inactivated fetal calf serum (FCS) (HCT116, HGT-1) or Ham F12/DMEM (v/v)/5% FCS (T84) or Ham F12/DMEM/10% FCS (SW48) or RPMI/10% FCS (Jurkat). All cell lines were mycoplasma-free.

### Cell treatment

Cell lines were seeded in 6-well plates at the following seeding density: 1.8 × 10^6^ cells per well for T84 and Jurkat; 1.5 and 1 × 10^6^ cells per well for SW48 and HGT-1, respectively; 1.2 × 10^6^ cells per well for HCT116. After a 3-day culture, proliferating cells were treated in presence or absence of HMW Poly-IC (0.64 μg/ml, *Invivo*Gen), a synthetic analog of dsRNA. In order to assess the effect of either extracellular or intracellular Poly-IC, cells were treated with Poly-IC alone or in combination with Dharmafect transfection reagent (3 μl/ml, Dharmacon, GE Health Care). Cells were incubated for 72 h or for various time periods in kinetic experiments (3, 6, 9, 12, 24, 48, 72, 96 h). Following Poly-IC treatment, supernatants were harvested for measurement of IFN-λ levels by ELISA. Cells were then detached with trypsin/EDTA (Life Technologies) and counted using a hemocytometer and the Trypan blue exclusion method. In some experiments, cells were treated with the cell-permeable peptide SN50 (80 μg/ml, Santa Cruz), known to inhibit NFκB nuclear translocation, or its inactive control peptide SN50M (80 μg/m, Enzo), with or without poly-IC for 18 hours.

### Clonogenic assay

To determine the clonogenic potential of the shed cells, detached cells of poly-IC treated (72 h) or control untreated cells were seeded in 75 cm^2^ flasks in fresh culture medium. After 11 days culture, colonies were counted under a binocular microscope (Nikon). Results are expressed as the percentage of the number of colonies divided by the number of cells seeded.

### IFN-λ ELISA

IFN-λ was measured in the cell supernatants using the IFN-λ 1-3 duoset ELISA (Biotechne), according to the manufacturer’s protocol.

### NFκBp65 translocation

The nuclear translocation of NFκB was assessed using immunofluorescence on T84 cells grown on 4 well glass chamber slides (BD Falcon culture slides, seeding density 322,000 cells per well).

Two days after seeding, cells were treated with 0.64 μg/ml poly-IC for 30 minutes, a period of time determined by preliminary time course experiments. After a pretreatment with triton X100 (Fluka; 0.1% in PBS for 5 min), and then with rabbit serum (Vector; 1.5% in PBS for 20 min), cells were incubated with a monoclonal antibody directed to NFκB p65 (Santa Cruz, SC-8008; 1:300 for 1 h) and then with alexa fluor A488 rabbit anti-mouse antibodies (InVitrogen, A11059; 1:300 for 1 h). Nuclei were stained with Dapi (1:1000, InVitrogen). Sections were mounted using Prolong anti-fade medium (InVitrogen). The fluorescence was observed on a fluorescent microscope equipped with an Apotome slider which eliminated image blurring (Axiovert 200-M, Carl Zeiss). Image processing was performed using AxioCam MR camera and Axiovision software (Zeiss).

### siRNA transfection

T84 cells were seeded on 6-well plates (700,000 cells per well). After a 24 h culture, siRNA RelA or the scrambled siRNA control (50 nM in Dharmafect) were added for 48 hours, according to the manufacturer’s protocol (Dharmacon siRNA transfection protocol). Cells were allowed to recover in fresh medium for 24 h and then treated with poly-IC for 18 h.

### Immunoblot analysis

For total proteins extraction, cultured cells’ pellets were homogenized in a stringent SDS-containing RIPA buffer containing protease inhibitors as described previously [23]. Protein concentration was determined using the Lowry assay (DC protein assay kit; Bio-Rad, Hercules, CA, USA). The protein-containing lysate (15 μg) was run on 10% SDS-polyacrylamide precast gels (Bio-Rad) and electrotransferred onto nitrocellulose membranes (Bio-Rad). After an overnight blocking (1% reagent; Roche Diagnostics), the membranes were probed with mouse monoclonal antibodies directed to NFκB p65 (SC 8008, 1:275, Santa Cruz), or β-actin (1:10,000, Sigma Aldrich) or with the polyclonal antibody MxA (1:1000, Sigma Prestige Antibodies), followed by horseradish peroxidase-conjugated goat anti-mouse or goat anti-rabbit antibody (1:2000, Santa Cruz and 1:10,000, Jackson, respectively). The immunoreactive proteins were detected on films using an enhanced chemiluminescence substrate according to the manufacturer’s instructions (Roche Diagnostics).

### Statistics

Each experiment was performed in triplicate. Results were expressed as mean ± sem. Statistical analyses were performed using a student *t*-test. A *p* value of less than 0.05 was considered significant.

## Abbreviations

DMEM: Dulbecco’s Modified Eagle Medium
dsRNA: double stranded RNA
EDTA: ethylenediaminetetraacetic acid
ELISA: enzyme-linked immunosorbent assay
FCS: Fetal Calf Serum
HMW: high molecular weight
IFN: interferon
NFκB: Nuclear Factor kappa B
NK: natural killer
siRNA: small interference RNA
